# Institutional Notes and News

**Published:** 1911-02-04

**Authors:** 


### Institutional Notes and News.

The Rev. Canon Hulton presided over the recent annual meeting of the governors of Lincoln County Hospital. A businesslike tabulation of bare facts and figures constituted the subject of the meeting, which presented nothing strikingly new. It is no contradiction of this to add that at Lincoln as elsewhere the numbers of out-patients have increased, with the inevitable result of pressing the existing accommodation beyond the limits for which it was intended. Mr. A. Moore, the secretary-superintendent, stated that the annual expenditure had gone down by £980 in the past twelve months, but this sudden drop was due to the redecoration of the wards a year ago, which had sent up the hospital's expenses in proportion. The balance due to the treasurer had amounted to about £190, and last year's income was £6,318, against an expenditure of £6,651. The daily average number of patients was 97.5. Several legacies are yet to come in, and the financial position on the whole is satisfactory.

**Lincoln County Hospital.**

The following resolution has been passed unanimously by fifty-seven medical practitioners of Newcastle and the district, who met to consider the question of hospital abuse: "That this meeting of medical practitioners of Newcastle and district respectfully requests the Admissions' Committee of the Royal Victoria Infirmary and the committee of the medical staff to consider the advisability of having a number of representative medical practitioners to confer with them on the question of hospital abuse, and the scheme to be adopted for the admission of patients." A committee of twelve has been appointed to carry out the resolution. This meeting, of course, is the latest phase of a situation of which readers of The Hospital have been informed fully in our previous issues of December 24, p. 385, and of January 14, p. 485.

**Victoria Infirmary, Newcastle.**

Mr. G. D. Woollcombe, the hon. secretary of the Newton Abbot Hospital and Dispensary, said, at the recent annual meeting, that during the past year they had had more patients than ever, and the hospital had done more work than it had ever done. This is certainly borne out by the report, which stated that in 1910 the number of patients rose to 410, while there were 575 out-patients and casualties; these latter had amounted to 617 in 1909. This hospital possesses also private wards, in which twelve patients had been treated, a great drop from the previous figure of 32. This has meant, of course, a smaller sum received, but against this must be set the slightly increased sum which has been received for private nursing. The total receipts of the hospital were £2,150, and the total expenditure was £2,095, leaving a balance of £55 odd in hand. The meeting was presided over by Mr. Edgcumbe Parson, the chairman of the house committee.

**Newton Abbot Hospital.**

Lord Balfour of Burleigh, who has done so much good work in raising the funds for the Queen Alexandra Sanatorium at Davos, Switzerland, presided last week at the annual meeting. At this meeting he showed that there is still much work to be done, and put forward an appeal for a special fund for the completion of the roads, paths, and drainage, which the site on the west side of the valley, 1,000 feet above the town, makes an expensive and necessary addition to the building. A laundry is also to be built, and the wants of the patients as regards books and periodicals are not to be forgotten. This last is not so unimportant as people in England may fancy, for the Sanatorium is not only a long way from the English town library, but the library of the town itself only two seasons ago received some severe criticism, and though the proposed appointment of the late Mr. William Lethbridge to the presidency of the library committee would have done much to remove the inertia that prevailed, his death only a few months later necessarily put a stop to his efforts. Among the other important speakers at the annual meeting were the Rev. E. S. Woods, the British Chaplain, Dr. W. R. Huggard, and Lord St. Vincent.

**Alexandra Sanatorium at Davos.**

The Cardiff Infirmary appeal has been conducted by energetic hands, and the latest success their efforts have achieved is the second festival dinner, over which Lord Tredegar presided last week. It is typical of the care which has been taken to bring the Cardiff Infirmary up to date and to keep it debt-free that the dinner was enlivened by amusing speeches. Hospital dinners are not always fortunate in this respect, but then Mr. Sydney Holland cannot dine everywhere. He was captured for Cardiff, however, and came out with one of his original suggestions for raising money, namely, the birthday brigade. For the benefit of hospital secretaries in need of a new idea for attracting subscriptions this may be defined as a band of people prepared to keep one bed on one day every year. The cost to them is five shillings and sixpence, and the day their own birthday. The last lingering doubt in anybody's mind about the desirability of subscribing to the Cardiff Infirmary as part of the National Memorial of Wales should be removed by Mr. Holland's support. He said: "As a hospital worker I wish to offer you a tribute of admiration for what you have done in Cardiff. You have changed your policy. You used to have a policy of restricting your work only to the money you had in hand. You did not then realise that the charity of the public is your capital which you can draw upon—which is safe if you do good work." He suggested also that the Infirmary should be rechristened the Royal Infirmary, Cardiff. Mr. Holland must have forgotten the decision recorded in The Hospital of October 22, 1910 (page 119), to rename the hospital King Edward VII.'s. This is a detail, however, and Colonel Bruce Vaughan must be congratulated, on behalf of the hospital's supporters, on the £2,800 which was subscribed during the dinner.

**Cardiff Infirmary.**

Clacton has one of the few hospitals the secretary of which is a woman, and decriers of the sex, therefore, are respectfully reminded that the hospital's chairman spoke in words of special praise of the work of Miss M. B. Baker. It should be added that the hospital's balance sheet reveals a balance on the right side of £43, the income being £343 and the expenditure £300. The number of patients treated was 86. The eleventh annual report of the Clacton and District Hospital is forced to record the death of several of its prominent supporters. Among them the name of Mr. John Smith will attract at once the notice of the hospital's friends, for he had been on the board of management since the foundation and had helped to guide it through more than one crisis in its career.

**Clacton Cottage Hospital.**

**Hereford Hospital and School Children.**

The strained relations of the Herefordshire General Hospital to the County Education Committee on the question of the medical treatment of inspected children was noted in The Hospital's Institutional Notes of January 21. Since then Sir Archer Croft, an important representative of the institution, speaking at the quarterly meeting of the County Council, mentioned the hospital's decision not to receive defective school children after June 30, and remarked that "the difficulty might be overcome by a grant or sale for" (not the hospital but) "the local medical practitioners. The Council inspected their roads and paid for them. It also inspected the school children, and ought to pay for them." Sir Archer had previously said he "hoped the hospital would never be thrown on the rates"! He trusted that those subscribers who had threatened to withdraw their subscriptions if the notice were carried out would not do so. This situation becomes instructive when compared with a motion brought forward by Mr. R. Bray at the L.C.C. Education Committee meeting on January 25, which read: "That inasmuch as the present method of assessing parents' payments in respect of medical treatment is causing (1) parents to be deprived of the free medical treatment for their children which they have hitherto received from the hospitals; (2) the overcrowding of those hospitals with which the Council has no agreement, and which, consequently, provide free treatment as before; the Children's Care (Central) Sub-committee do forthwith submit proposals which will insure that they will be required to contribute only in those cases when the hospitals decide that their circumstances are such that free medical treatment should be refused." Mr. Bray is reported to have said that there was growing indignation among poor parents against having to pay for the treatment of their children at hospitals which hitherto had been free to them. This indignation would certainly react upon the Hospital Saturday and Sunday Funds, which the working classes up to now had supported.

**Moreton Hampstead Cottage Hospital.**

An interesting year's work was disclosed at the recent annual meeting of the Moreton Hampstead Cottage Hospital. Two new rooms were added to the institution during the year, and for the gift of the labour necessary for this work the hospital has to thank the Hon. F. W. D. Smith, its president, who took the chair at the annual meeting. It is true that the year ended with a debt of £40, but this is rather proof of the increased work and popularity of the institution, of which the new rooms and the more than adequate subscriptions towards this construction are the visible signs. Fifty-three patients were attended in the wards, and each had an average stay of 20.7 days. There were 19 out-patients. The balance on the building fund mentioned above was, on Mr. Smith's suggestion, placed on deposit at the bank. The balance sheet was presented by Mr. E. S. Cuming, the hon. secretary.

**The Melbourne Hospital.**

The annual report of the Melbourne Hospital for the year ended June 30, 1910, is to hand, from which it appears that the total number of patients treated was 23,644, including 6,162 in-patients and 17,482 out-patients and casualty cases. The average daily number of in-patients treated was 320.7, and the average stay of each patient in the wards was 19 days. No fewer than 2,160 anæsthetics were administered, of which 270 were local, and four spinal anæsthetics. The most usual general anæsthetic employed was ether, after induction by ethyl chloride. There were no fatalities from anæsthetics during the year. The death-rate for the year was 12 per cent., there being 175 deaths within 48 hours of admission, and 562 other deaths. The hospital had to cope with a severe outbreak of typhoid fever in the summer, there being no fewer than 70 of these patients in the hospital at the same time. The ordinary income of the hospital was £26,325, against an ordinary expenditure of £30,448. The average cost per bed occupied was £78 2*s*. 6*d*., the cost per patient averaging £4 1s. 3d. Lovers of statistics will find in these figures some food for meditation, but as such are rare we do not indulge them often with so many, unrelieved as these are by illustration or comment.

**Leed's Women's Hospital.**

It is interesting to learn how the co-operation which the Leeds Women's Hospital has undertaken during the past year with the general infirmary and the public dispensary of the town by means of the lady almoners has been working. So satisfied are the Women's Hospital authorities with the value of this work that it is to be continued, and the annual report contains an important statement for which the lady almoners are responsible, which is that "the services of the medical men at the institutions mentioned would be much more efficient if they were followed up in the homes of the patients by the almoners and their voluntary and trained workers." This is an instructive practical illustration of the truth of the contentions which The Hospital published on the subject on July 2 and 23, 1910. It is discouraging to add that this good work has been accompanied by a deficit of £980. Mr. F. R. Spark remarked at the annual meeting that the Leeds General Infirmary threatened to absorb all the available subscriptions in its appeal for £150,000 to reconstruct itself, and as it was suggested to call this Fund a memorial of the whole city to King Edward, he suggested, as a member of the Leeds General Infirmary Board, that at least 10 per cent. should be devoted to the other medical charities of the city. In-patients numbered 859, and 16,410 children and women have been treated in the out-patient department.

**Leicester Homœopathic Cottage Hospital.**

The Homœopathic Cottage Hospital at Leicester congratulates itself in the report on the "brilliant results achieved" in many of the 52 cases treated in its wards during the past twelve months. Indeed, the following passage in the report is worthy of quotation: "The list of grateful patients, keenly appreciative of private treatment at a small cost instead of in the public wards of a large infirmary, was increasing year by year, and the committee were bound once more to emphasise the important fact that public funds were thus saved, the patients at the same time having the advantage of homœopathic methods and maintaining their independence. Unfortunately, fatal termination occurred in two cases, but in each the result was inevitable." There is a spirit in this passage which is worth noticing, and an enthusiasm which has turned what is too often the dullest of the year's documents, a hospital report, into something more like life and literature. We quote in order to show that it is the spirit of the compiler, and not the spirit of the subject, which causes too often reports of hospitals to remain unread. If dull, they deserve to be so. This hospital's finances have improved, an adverse balance of £58 having been reduced to one of £17 in the course of the year. Owing to insufficient support of homœopathy at Leicester the cherished plan of removing the hospital to another site has been abandoned for the present. Those disappointed at this will be encouraged, perhaps, by reading certain remarks on restrictive finance made at a festival dinner at Cardiff, and recorded in our Note on that institution in this issue.

